# Microstructure and Dielectric Properties of Gradient Composite Ba_x_Sr_1−x_TiO_3_ Multilayer Ceramic Capacitors

**DOI:** 10.3390/mi15040470

**Published:** 2024-03-29

**Authors:** Xiaobing Jili, Libin Gao, Hongwei Chen, Jihua Zhang

**Affiliations:** 1 School of Electronic Science and Engineering, University of Electronic Science and Technology of China, Chengdu 610054, China; jilixiaobing@163.com (X.J.); hwchen@uestc.edu.cn (H.C.); 2 State Key Laboratory of Electronic Thin Films and Integrated Devices, University of Electronic Science and Technology of China, Chengdu 610054, China

**Keywords:** multilayer ceramic capacitors (MLCCs), Ba_1−x_Sr_x_TiO_3_ (BST), tunable capacitors

## Abstract

Multilayer ceramic capacitors (MLCCs) prepared using Ba_1−x_Sr_x_TiO_3_ (BST) ceramics exhibit high dielectric constants (~1000), low dielectric loss (<0.01), and high breakdown voltage, with particularly significant tunability in dielectric properties (>50%) and with poor temperature stability. Doping-dominated temperature stability improvements often result in unintended loss of dielectric properties. A non-doping method has been proposed to enhance the temperature stability of BST capacitors. The composite gradient multilayer (CGML) ceramic capacitors with Ba_x_Sr_1−x_TiO_3_, where 0.5 < x < 0.8, as the dielectric, were prepared using a tape-casting method and sintered at 1250 °C. There exists a dense microstructure and continuous interface between the Ba_x_Sr_1−x_TiO_3_ thick film and the Pt electrodes. CGML ceramic capacitors feature a high dielectric constant at 1270, a low dielectric loss of less than 0.007, and excellent frequency and temperature stability. The capacitor showcases remarkable dielectric properties with a substantial tunability of 68% at 100 kV/cm, along with a notably consistent tunability ranging from 20% to 28% at 15 kV/cm across temperatures spanning from 30 to 100 °C, outperforming single-component BST-MLCCs in dielectric performance.

## 1. Introduction

Amidst the dynamic evolution of electronics technologies, multilayer ceramic capacitors (MLCCs) have garnered extensive utilization in diverse microelectromechanical systems owing to their substantial capacity and unwavering reliability [1]. Moreover, for the advancement of high-performance power devices, forthcoming MLCCs must exhibit outstanding temperature stability, a high dielectric constant (ε_r_), substantial capacitance tunability, and a reduction in size through miniaturization [1].

Barium strontium titanate (Ba_x_Sr_1−x_TiO_3_) is a perovskite-type ferroelectric material known for its high dielectric constant, low dielectric loss, and tunable dielectric constant. The Curie temperature (T_C_) varies according to x as follows: T_C_ = (360x + 40) °C [2]. By changing the doping content of strontium in perovskite materials, the dielectric properties of BST materials can be effectively altered [3]. Thus, the utilization of MLCCs with BST material in microelectromechanical systems enables rapid adaptation to diverse and intricate operational environments. Although BST ceramic materials, epitomized by Ba_0.6_Sr_0.4_TiO_3_, exhibit commendable dielectric tunability, attaining consistently high tunability across an extensive temperature spectrum remains a formidable hurdle [2,4]. 

Another class of extensively investigated tunable dielectric ceramic materials includes Bi_1.5_MgNb_1.5_O_7_/Bi_1.5_ZnNb_1.5_O_7_ (BMN/BZN) compounds, renowned for their superior temperature stability and minimal losses. However, they have a smaller dielectric constant compared to BST (~200), and there is a lack of effective methods to increase it. It is difficult to observe tunable dielectric properties in BMN materials, apart from BMN thin film, and the tunability of the dielectric constant is relatively low (30% at 1.5 MV/cm) [5]. Furthermore, the volatility of Bi/Mg elements poses challenges to the stability of ceramic properties.

Moreover, organic material-based tunable dielectrics exhibit characteristics such as flexibility, light weight, and ease of processing. However, the limited dielectric constant of organic materials, their lower operating frequencies, and the sensitivity of polymer materials to environmental factors make them less suitable for microwave applications [6].

According to the temperature range and capacitance variation characteristics of MLCCs, ceramic capacitors are commonly categorized as X5R, X7R, Y5V, C0G, and similar types. Among these, X7R is identified as a type of temperature-stable ceramic capacitor, where the capacitance variation remains within a narrow 15% range across temperatures ranging from −55 °C to +125 °C. The Sr-doped BaTiO_3_-based X7R ceramic prepared by Hai W et al. has a dielectric constant of about 2000 and temperature coefficients of capacitance at 125 °C of 11% [7]. However, elevated doping levels correspondingly precipitate a marked diminution in the dielectric tunability of BST materials [2]. Another effective way to augment the temperature stability of MLCC is utilizing a core–shell structure, which can result in frequency dispersion, consequently subject to certain limitations [8,9]. Should a material emerge that is capable of ensuring temperature stability alongside high tunability, a high dielectric constant, low losses, and superior frequency stability, it would herald a significant advancement for MLCCs and the broader field of electronics.

In recent years, researchers have uncovered unique properties within gradient ferroelectrics previously unseen in homogeneous-bulk or thin-film ferroelectrics [10]. The spatial variance of electric dipole moment delineates gradient ferroelectrics from their homogeneous counterparts [11]. Owing to interlayer electrostatic and electromechanical interactions, gradient structures exhibit enhanced dielectric properties in comparison to homogeneous monolithic bodies [12]. Structures featuring compositional gradient alterations can effectively mitigate device dielectric loss, broaden the temperature range for stable device operation, and prove highly effective in applications such as tunable antennas [13]. Multilayer thick films with composite gradient compositions, prepared using doped BST, showcase reduced dielectric loss and an expanded stable temperature range for dielectric tuning [14]. As the Sr content increases, the Tc of BST ceramics undergoes a decline, resulting in a decreased dielectric constant and less dielectric loss in RT [15]. Choi et al. achieved high dielectric response (~2000), tunability (51% at 20 kV/cm), and temperature stability over a wide temperature range (30–90 °C) by designing a composite gradient multilayer (CGML) structure [16]. Furthermore, it was discovered that compositionally graded MLCC exhibits enhanced dielectric tunability (70%) and low dielectric loss (<2.5%) [17]. Given the notable tunability of BST materials’ dielectric properties near the Tc [16], employing BST materials with substantial composition variation (Ba_x_Sr_1−x_TiO_3_, where x = 0.5, 0.6, 0.7, 0.8) as the dielectric layer for MLCCs offers a promising approach to achieve stable high dielectric tunability, a high dielectric constant, and low dielectric loss across a broad temperature range.

Despite the clear conclusions reached regarding the performance of BST ceramics, the transfer of dielectric materials to multilayer components remains a significant challenge [18]. Establishing a stable and continuous interface between the metal and dielectric layers proves to be a critical factor in the fabrication of MLCC during the co-fired process [19]. The first challenge encountered is how to choose the metal for the internal electrodes of BST-MLCC. Nickel is often used as the internal electrode for MLCCs due to its low cost and resistance to reactions with ceramics [20]. However, when using nickel metal as the internal electrode during co-firing, the sintering process must be carried out in a reducing atmosphere. Otherwise, the nickel metal will oxidize into nickel oxide, significantly increasing the internal resistance of the MLCC and consequently compromising its performance. Unfortunately, in a reducing atmosphere, due to the defective chemical nature of the dielectric ceramics, in order to prevent semiconductor transformation after firing in a reducing environment, it is necessary to perform net acceptor doping on the dielectric ceramics [21]. Additionally, it should be noted that when using Ag_0.7_/Pd_0.3_ as the internal electrode co-fired with high-purity strontium barium titanate at high temperatures, the electrode metal tends to permeate into the dielectric layer, resulting in the loss of the multilayer structure of the MLCC [22]. Due to the stable chemical properties of Pt, which make it less prone to reacting with other gases during the sintering process, and the fact that its sintering temperature aligns well with that of high-purity strontium barium titanate [22], pure Pt was chosen as the internal electrode for BST-MLCC. 

In this study, high-purity Ba_x_Sr_1−x_TiO_3_ thick films, where 0.5 < x < 0. 8, were utilized to fabricate CGML capacitors at high temperatures (>1200 °C). In order to facilitate a more straightforward comparison of CGML capacitor performance, pure Ba_0.8_Sr_0.2_TiO_3_ and Ba_0.5_Sr_0.5_TiO_3_ were chosen as the dielectric layers for the MLCC, with all other preparation conditions being consistent with CGML capacitors. In comprehensive comparison, the CGML capacitor composed of Ba_x_Sr_1−x_TiO_3_ thick films, where 0.5 < x < 0.8, exhibits high dielectric tunability and low dielectric loss. Furthermore, it exhibits a dielectric tunability ranging from 22% to 29% (at an electric field strength of 15 kV/cm) within the temperature interval of 20 °C to 100 °C. Conversely, the tunability of single-component BST-MLCC diminishes substantially from 35% to 12% over the same temperature range.

## 2. Experimental Procedures

**Figure 1:**
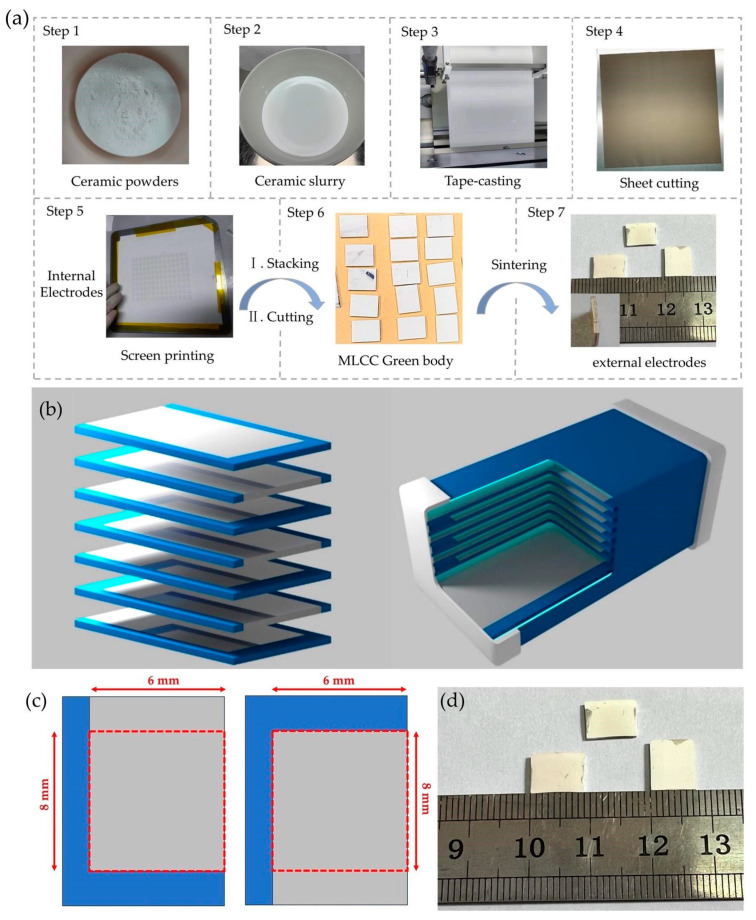
(**a**) The diagram illustrating the fabrication process of the MLCC. (**b**) Schematic diagram of the BST-MLCC structure. (**c**) Schematic diagram of the MLCC inner electrode. (**d**) The finished MLCC with an area of 10 mm × 6 mm.

The fabrication process of the MLCC is illustrated in Figure 1a. Utilizing the gradient variation in Ba/Sr composition, we prepared high-purity Ba_x_Sr_1−x_TiO_3_ (x = 0.5, 0.6, 0.7, 0.8) ceramic powders through the solid-state reaction sintering method. The powders were blended with organic solvents in specific ratios and subsequently ball-milled to produce ceramic slurries exhibiting excellent flow characteristics. Subsequently, the ceramic slurry was transformed into defect-free BST ceramic thick films through tape casting, and were subsequently sliced into uniform sheets. Following that, inner electrodes were screen-printed and laminated alternately with BST thick films. During the stacking process, ceramic protective layers were added to the top and bottom surfaces to increase mechanical strength and enhance insulation performance. The stacked bars were enclosed in laminating bags and vacuum-sealed. Pressure was then applied using isostatic pressing to ensure tight and solid bonding between the layers within the bars. The laminated bars were cut into individual MLCC green bodies. In the final stage of the process, the green bodies undergo high-temperature sintering, transforming them into ceramics. The outer electrodes were then applied, followed by the necessary processing steps, ultimately resulting in the final assembly of the MLCCs.

In this paper, Ba_x_Sr_1−x_TiO_3_ ceramic powders, where 0.5 < x < 0.8, were prepared using the conventional solid-phase reaction method with raw materials: analytical grade oxides BaCO_3_ (B112362, Aladdin, Shanghai, China), SrCO_3_ (S102039, Aladdin, Shanghai, China), and TiO_2_ (T299213, Chron Chemicals, Chengdu, China) in stoichiometry. The BaCO_3_, SrCO_3_, and TiO_2_ powders were mixed according to the calculated proportions (four different Ba_x_Sr_1−x_TiO_3_ ceramic powders are required, with x = 0.5, 0.6, 0.7, 0.8). The mixed powders were added to ethanol and ball-milled for 24 h using a planetary ball mill (PM100, Retsch, Germany). The powder mixture was further ball-milled with ethanol for an additional 24 h. After drying the ethanol at 90 °C, the dried powder was pre-sintered at 1200 °C for 2 h. The post-sintered powder product was then subjected to another 24 h of ball milling. Ba_x_Sr_1−x_TiO_3_ thick films, where 0.5 < x < 0.8, and the MLCCs were manufactured using the cast tape method, and their structure is shown in Figure 1b. The powder obtained from the secondary ball milling and drying steps was added to a suitable mixture of toluene and ethanol to create a slurry for tape casting. In addition, polyvinyl butyral (PVB) (30153960, Sinopharm Chemical Reagent Co., Ltd., Shanghai, China) was employed as the binder, and dioctyl phthalate (DOP) (D109646, Aladdin, Shanghai, China) served as the plasticizer, both mixed through ball milling to disperse the powder in the solvent. A BST thick film of 150 mm × 150 mm was manufactured by the tape casting machine (HXL Y-011, Zhaoqing Huaxinlong Automation Equipment, Zhaoqing, China), as shown in Figure 1a. To fabricate the MLCCs, Pt inner electrodes (02H-2030D, Sryeo Paste, Shenzhen, China) with an effective area of 8 × 6 mm, were staggered onto BST tape using a screen printer (HXSY-012, Zhaoqing Huaxinlong Automation Equipment, Zhaoqing, China), as shown in Figure 1a–c. Subsequently, as shown in Figure 1b, eight pieces of Ba_x_Sr_1−x_TiO_3_ tapes, where 0.5 < x < 0.8, were stacked together using a laminator (HXYT-013, Zhaoqing Huaxinlong Automation Equipment, Zhaoqing, China) at 50 °C and 14 MPa of pressure. The laminated BST green bodies were prepared by isostatic pressing at 65 °C and 20 MPa for 30 min (H8305520, Honqchi, Kunshan, China). Burn-out of the organic material was conducted at 400 °C with a heating rate of 0.33 °C/min to remove all solvents and adhesives. The BST-MLCC and CGML capacitor were sintered at 1300 °C, 1250 °C, and 1200 °C for 1.5 h. After sintering, both ends of the MLCC and CGML were coated with high-temperature (850 °C) silver paste as the outer electrode, as shown in Figure 1a,d, and the finished size was about 9 mm × 7 mm. 

The phase structure of the crystalline samples was characterized by X-ray diffraction (XRD, BeDe D1) with CuKα radiation. Observation of the surface morphology of the crystal samples was conducted using a scanning electron microscope (SEM, HITACHI, S-3400N, Shenzhen, China). Wayne Kerr 6500P (Wayne Kerr, Shenzhen, China) measured the temperature dependence of the dielectric properties of the samples at 20–200 °C and 100 kHz. The variation in $\varepsilon_{r}$ and tanδ with frequency was measured using Agilent 4294A (Keysight, Santa Rosa, CA, USA) at 1 kHz to 1 MHz, where the $\varepsilon_{r}$ of the MLCC samples was calculated with a dielectric layer thickness of 30 μm and an effective electrode area of 384 mm^2^. The tunable characteristics of the specimens were studied using a Semiconductor Testing and Analysis System (PST6747A, Bontec, Beijing, China). The tunability of the dielectric constant in the MLCCs was derived from the following equation:

(1) $$\mathrm{Tunability}\;(\%)=\frac{\varepsilon_{2}{-\varepsilon }_{1}}{\varepsilon_{2}}\times 100\%$$

where the $\varepsilon_{1}$ is the dielectric constant of the dielectric at $V_{1}$ electric field ($V_{1}$ = 100 kV/cm), and $\varepsilon_{2}$ is the dielectric constant measured at $V_{2}$ electric field ($V_{2}$ ≈ 0 kV/cm). 

## 3. Results and Discussion

**Figure 2:**
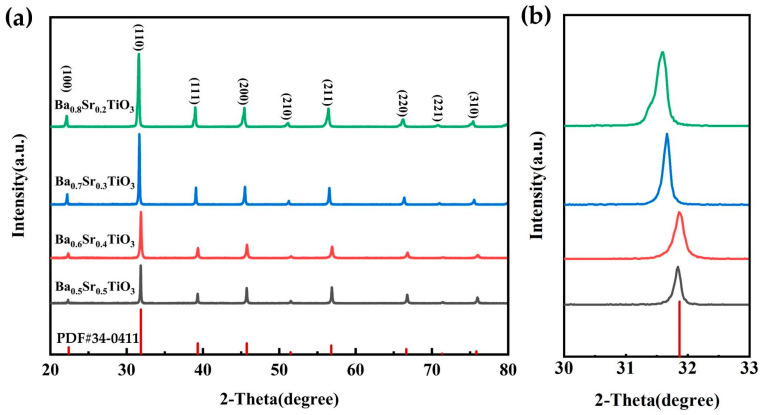
(**a**) XRD patterns of BST thick film samples with different Ba/Sr rations. (**b**) Magnified portion of the main peak section in the XRD pattern.

Ba_x_Sr_1−x_TiO_3_ ceramics, where 0.5 ≤ x ≤ 0.8, were calcined at 1200 °C for 4 h and they evinced a pure perovskite phase, as displayed in Figure 2a. As shown in Figure 2b, it can be observed that with the increase of x from 0.5 to 0.8, the main peak of the pre-sintered Ba_x_Sr_1−x_TiO_3_ powder shifts to the left at 31.6°. 

The variation in this phenomenon is attributed to the decrease in lattice parameters of the pre-sintered powder, which occurs as the Sr^2+^ ion concentration increases, owing to the smaller ionic radius of Sr^2+^ ions (1.44 Å) compared to Ba^2+^ ions (1.61 Å) [23]. All lattice parameters are shown in Table 1. Additionally, the crystallinity of all BST powder samples is above 95% which is calculated as the percentage contribution of the crystalline portion to the total area under the peaks of X-ray diffraction intensity for the crystalline polymer [16]. Thus, the prepared powder is in accordance with expectations.

**Figure 3:**
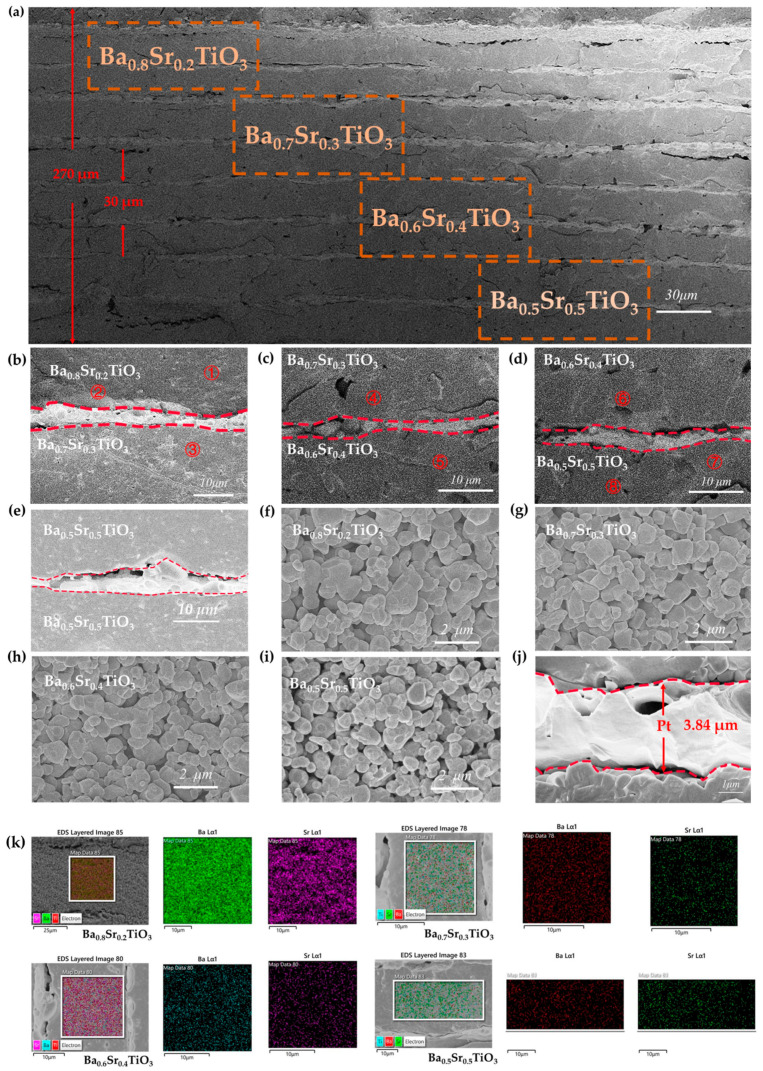
SEM image of the CGML capacitor prepared at 1020 °C (**a**), interface between layers (**b**–**e**), Ba_x_Sr_1−x_TiO_3_ (x = 0.8, x = 0.7, x = 0.6, 0.5) grains (**f**–**i**), metal platinum internal electrode (**j**). (**k**) SEM/EDS of dielectric layers with different compositions.

As shown in Figure 3a, the SEM image of the MLCC is composed of eight layers of Ba_x_Sr_1−x_TiO_3_ thick films, with two layers in the same components. It has a structure consistent with Figure 1b. The MLCC was sintered at 1250 °C, and Pt was used as the electrode for this device. The eight layers of Ba_x_Sr_1−x_TiO_3_ thick films are well sintered in the MLCC without any fractures. It can be clearly seen that there is a distinct and clear interface between the metal and dielectric layers, indicating a good compatibility between the BST dielectric and Pt metal. The total thickness of its internal multilayer capacitor structure was ~270 μm, with the thickness of the BST thick film being approximately 30 μm, and the thickness of the Pt electrode being approximately 3.5 μm. The pores at the interface may be caused by the uneven shrinkage rates of BST thick films with different compositions during the sintering process.

The enlarged images of the interfaces between the BST and different compositions are depicted in Figure 3b–e. The interface between the thick film and the electrode exhibits a continuous and uniform structure. Figure 3k illustrates the SEM/EDS results of the dielectric layer with different compositions. The elements Ba, Sr, and Ti are evenly distributed throughout the dielectric layer. Across the four different compositions of the dielectric layer, the Ti content remains relatively consistent. With an increase in Sr content, the distribution map of the Sr exhibits a denser pattern, whereas the distribution map of the Ba suggests a more scattered distribution. This highlights the notable compositional variations within the dielectric layer. The corresponding elemental composition results from the EDS scans can be found in Table 2. In the EDS scan, the signal of the Ba element decreases with diminishing x, while the signal of the Sr element increases with decreasing x. The substantial gradient difference between distinct layers indicates a virtually negligible impact between them. The EDS scan from distinct regions within the same layer demonstrates a relatively consistent elemental composition across various areas. This affirms the development of a stable and uniform thick film within the dielectric layer, free from the influence of other layers during the sintering process.

The microstructure of the Ba_x_Sr_1−x_TiO_3_ thick films in the CGML capacitors sintered at 1250 °C was analyzed through SEM analysis, as shown in Figure 3f–i. The thick films of BST with diverse compositions preserve their unique characteristics. All components of the thick films exhibit dense microstructures with virtually no pores. When x = 0.8, the grain size is approximately 2 μm, as shown in Figure 3f. Additionally, with the increase in Sr^2+^ content, the grain size gradually decreases. When the Sr^2+^ content added reaches 50%, the grain size of the thick film reaches approximately 0.6 μm, and the pore size of the thick film increases. The electrode layer exhibits a dense structure and a distinct and well-defined boundary is formed between the electrode layer and the dielectric layer, as shown in Figure 3j. The thickness of the internal metal electrode is approximately 3.84 μm.

**Figure 4:**
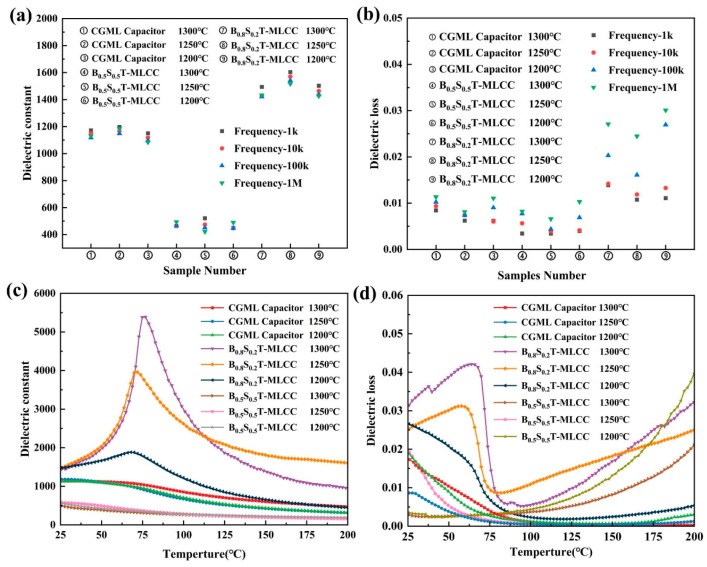
Scatter plot of the dielectric constant (**a**) and dielectric loss (**b**) of MLCCs and CGMLs with variety frequency. The temperature dependence of $\varepsilon_{r}$ (**c**) and tanδ (**d**) of the MLCC and CGMLs.

The dielectric constant of the BST-MLCC sintered at 1300 °C, 1250 °C, and 1200 °C, varied with the measurement frequency, as shown in Figure 4a. The dielectric constant of the CGML capacitor is approximately 1200, surpassing that of the BST (0.5/0.5)-MLCC (~500), and the difference is relatively small compared to the BST (0.8/0.2)-MLCC with the highest dielectric constant (~1550). This may be attributed to the fact that the Curie temperature of the CGML capacitors is closer to room temperature compared to the BST (0.5/0.5)-MLCC. Along with the rise in frequency, the dielectric constant of the CGML capacitors experiences a slight decrease, while other samples vary significantly. This evidence suggests that ceramic capacitors fabricated using the CGML structure do not manifest a conspicuous decline in their frequency stability.

The dielectric loss of various samples is illustrated in Figure 4b at frequencies of 1 kHz, 10 kHz, 100 kHz, and 1 MHz. The dielectric losses of all the samples are slightly increase with increasing frequency. The CGML capacitors with gradient variation and BST (0.5/0.5)-MLCC, which were sintered at 1250 °C, exhibits low dielectric loss, ≤0.008 at 1 MHz. The dielectric loss of the BST (0.8/0.2)-MLCC is relatively high (>0.01), and it increases with the rise of frequency. Moreover, the CGML capacitors exhibit superior microstructure and fewer pores, which could also be a reason for the lower dielectric loss. The lower dielectric loss endows CGML capacitors with a broader range of applications.

Dielectric constant variation in the BST-MLCC occurs with temperature in the range of 25 °C to 200 °C, as shown in Figure 4c. This was measured at 100 kHz. The dielectric constant of the BST (0.8/0.2)-MLCC exhibits an initial increase followed by a subsequent decrease with rising temperature. The Tc of the BST (0.8/0.2)-MLCC sintered at 1300 °C is around 80 °C. The Curie temperature exhibits a declining trend with the reduction in sintering temperature of the samples. At a sintering temperature of 1250 °C, the Curie temperature of the BST (0.8/0.2)-MLCC is around 75 °C. Where the testing temperature exceeds 25 °C, the Tc of CGML capacitors cannot be observed, possibly because its Curie temperature is below room temperature. Additionally, compared to the BST (0.5/0.5)-MLCC, the CGML capacitor exhibits a larger dielectric constant. This provides evidence that the Curie temperature of CGML capacitors significantly exceeds that of BST (0.5/0.5)-MLCCs. With the increase in testing temperature, they demonstrate better temperature stability compared to BST (0.8/0.2)-MLCC, as shown in Figure 4c.

Dielectric lose variation in the BST-MLCC with temperature in the range of 25 °C to 200 °C was measured at 100 kHz, as shown in Figure 4d. The CGML capacitors demonstrate a relatively low dielectric loss. The dielectric loss of CGML capacitors sintered at 1250 °C is only 0.008 at room temperature. Moreover, along with the increase in temperature, the dielectric loss significantly decreases further (≤0.005 at 100 °C). The decrease in dielectric loss with rising temperature can be attributed to the absence of domain walls in the paraelectric state [23]. The dielectric loss of the BST (0.8/0.2)-MLCC decreased at Tc, then it increased with temperature. An unmistakable phenomenon arises: despite the temperature significantly exceeding the Curie temperature, as the temperature rises, CGML capacitors exhibit superior dielectric loss and temperature stability compared to single-component MLCCs.

**Figure 5:**
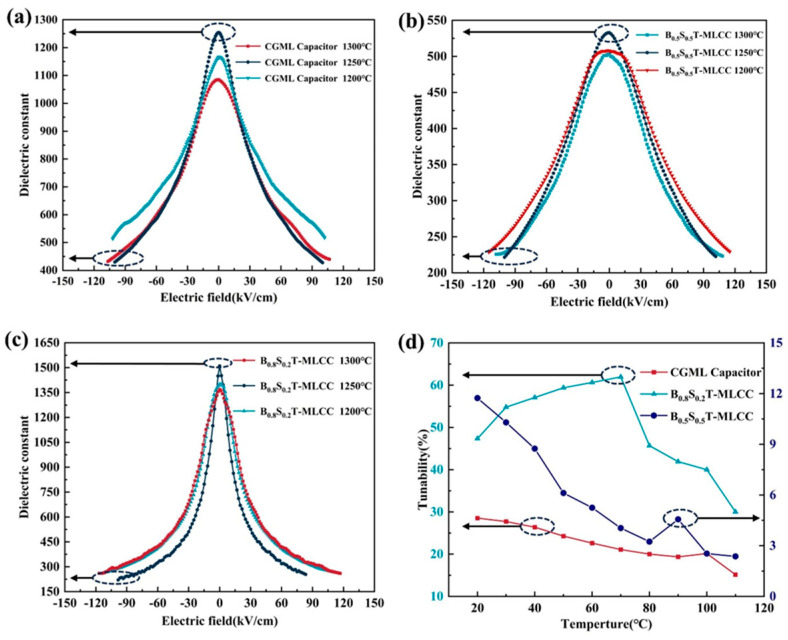
Variation in $\varepsilon_{r}$ value of a CGML capacitor (**a**), BST (0.5/0.5)-MLCC (**b**), and BST (0.8/0.2)-MLCC (**c**) with respect to the applied electric field at 100 kHz and tunability at 10 kV/cm with respect to the temperature of the BST-MLCC sintered at 1250 °C (**d**).

The dielectric constant of the BST-MLCC changes with the variation in electric field strength measured at 100 kHz, as shown in Figure 5a–c. Due to the significant difference between room temperature and Tc, all MLCC samples exhibit relatively low dielectric constants. The dielectric tunability of the MLCCs prepared under various conditions was tested at a frequency of 100 kHz and an electric field strength of 100 kV/cm, as shown in Table 3. The CGML capacitors sintered at 1250 °C demonstrate relatively superior dielectric tunability, as shown in Figure 5a. The dielectric tunability of CGML capacitors is approximately 68.85%. Additionally, their breakdown field strength is also higher. The BST-MLCCs sintered at 1300 °C exhibit high breakdown strength. Although the dielectric loss of the BST (0.5/0.5)-MLCC is relatively low (≤0.004 at 100 kHz), its dielectric tunability is relatively low (≤55%) compared to the CGML capacitors. The BST (0.8/0.2)-MLCC exhibits a relatively high dielectric tunability (86%), as shown in Figure 5c, but has severe dielectric losses compared to the CGML capacitors. 

The dielectric tunability of the BST-MLCC exhibits variation with increasing temperature, as shown in Figure 5d. The tests were conducted at a frequency of 100 kHz under an applied electric field strength of 15 kV/cm. At a testing temperature of 30 °C, the dielectric tunability of the BST (0.8/0.2)-MLCC is measured at 47%. With increasing temperature, the dielectric tunability of the BST (0.8/0.2)-MLCC gradually rises, reaching its peak near the Curie temperature, and subsequently decreases. As the temperature rises, the tunability of CGML capacitors and the BST (0.5/0.5)-MLCC gradually decreases. At the same testing temperature, the CGML capacitor exhibits a higher dielectric tunability compared to the BST (0.5/0.5)-MLCC. As the temperature increases, the decrease in dielectric tunability of CGML capacitors is more gradual. The dielectric tunability of the CGML capacitors remains more stable with increasing temperature compared to the other components. As the Tc of CGML capacitors is higher than that of the BST (0.5/0.5)-MLCC, this could explain the observed differences. The dielectric tunability of ferroelectric materials typically reaches its maximum near the Tc or the ferroelectric phase transition temperature. The use of Ba_x_Sr_1−x_TiO_3_ thick films with varying compositions in the preparation of CGML capacitors expands their stable and tunable temperature range.

The Curie temperature of the Ba_0.8_Sr_0.2_TiO_3_ material is approximately 70 °C, and that of Ba_0.7_Sr_0.3_TiO_3_ is around 40 °C [24]. They exhibit a ferroelectric phase at room temperature, demonstrating high dielectric constants and relatively high dielectric tunability [25]. With increasing Sr content in BST, the Curie temperature decreases, falling below room temperature when the Sr content exceeds 35%, exhibits a paraelectric phase at room temperature, showing good temperature stability of the dielectric constant and relatively low dielectric loss [26]. As a result, the Curie temperature distribution of Ba_x_Sr_1−x_TiO_3_ (0.5 < x < 0.8) thick films is distributed above and below room temperature. As shown in Figure 4c, for CGML capacitors, compared to single-component MLCCs, their Curie peaks are becoming flatter. In the absence of additional doping, they exhibit characteristics similar to relaxor ferroelectrics. As shown in Figure 5d, the tunability of CGML capacitors is also more stable with temperature variations. Furthermore, CGML capacitors have achieved a high dielectric tunability compared to BST (0.5/0.5)-MLCCs, as illustrated in Figure 5a–c. While doping broadens the Curie peak, the dielectric tunability decreases sharply with increasing doping ratio [2]. The presence of BST thick films with different compositions leads to the manifestation of the paraelectric phase in the overall CGML capacitor. As shown in Table 4, the dielectric constant and loss of CGML capacitors show slight decreases compared to single-component MLCCs and BST samples documented in other literature. However, CGML capacitors exhibit a notable improvement in temperature stability while still preserving a considerable degree of dielectric tunability. It is expected to be possible to obtain CGML capacitors suitable for different working environments by adjusting the composition of the thick film. 

## 4. Conclusions

The integration of micro-mechanical systems is effectively improved by using compact-size tunable MLCCs, reducing package size. Temperature- and frequency-stable capacitors make micromechanical systems under complex operating conditions possible. This means that developing reliable, highly tunable MLCCs is essential for advancing electronic systems. In this paper, BST-MLCCs and CGML capacitors were fabricated using the tape casting method under different sintering conditions. This CGML capacitor employs eight layers of Ba_x_Sr_1−x_TiO_3_ thick film with 0.5 ≤ x ≤ 0.8, with each composition comprising two layers. The CGML capacitor and MLCCs sintered at 1250 °C exhibit the formation of dense microstructure thick films without pores, all Ba_x_Sr_1−x_TiO_3_ thick films exhibit a dense and continuous interface with the Pt electrode. The SEM/EDS results indicate that the various BST thick films are independent of each other. With the decline in Sr content, a leftward shift is observed in the XRD results of BST thick films, accompanied by an increase in ceramic grain size. The CGML capacitor exhibits relatively good frequency stability, a relatively large $\varepsilon_{r}$ value of 1200, and a low dielectric loss of 0.0076. The CGML capacitor exhibits a dielectric tunability of 68.85% at 100 kV/cm and stable tunability (28–20% at 15 kV/cm) at temperatures ranging between 30 and 110 °C while that of BST (0.5/0.5)-MLCC decreases sharply (11–3% at 15 kV/cm). The range of the stable tunable temperature range can be expanded by employing Ba_1−x_Sr_x_TiO_3_ thick films with different compositions in the preparation of ceramic capacitors. 

## Figures and Tables

**Table 1:** The lattice constants and crystallinity of BST powder samples under different Ba/Sr ratios.

Samples	Ba_0.5_Sr_0.5_TiO_3_	Ba_0.6_Sr_0.4_TiO_3_	Ba_0.7_Sr_0.3_TiO_3_	Ba_0.8_Sr_0.2_TiO_3_
Lattice constants (Å)	3.9471	3.9650	3.9724	3.9869
Crystallinity	98.20%	95.02%	96.95%	95.16%

**Table 2:** EDS scan of the Ba_x_Sr_1−x_TiO_3_ thick film, where 0.5 ≤ x ≤ 0.8.

Spectrum	Ba (at.%)	Sr (at.%)	Ti (at.%)
①	40.73	12.71	46.65
②	40.67	12.65	47.15
③	35.04	17.65	47.31
④	35.12	17.35	45.46
⑤	29.13	21.36	47.02
⑥	29.24	21.46	46.62
⑦	24.27	28.89	46.84
⑧	24.98	26.15	46.98

**Table 3:** The dielectric tunability of BST-MLCCs prepared under different conditions (at 100 kHz, 100 kv/cm).

Samples	MLCC								
Sintering (°C)	1300	1250	1200	1300	1250	1200	1300	1250	1200
Ba/Sr	0.5/0.5	0.5/0.5	0.5/0.5	0.8/0.2	0.8/0.2	0.8/0.2	Gradient	Gradient	Gradient
Tunability (%)	53.13	57.49	52.07	79.18	85.48	79.94	58.79	68.85	55.62

**Table 4:** The dielectric performance of CGML capacitors compared to other capacitors.

Samples	This Work	Reference [16]	Reference [17]	Reference [27]	Reference [28]	Reference [29]
Dielectric Constant	1270	~2000	~2000	102	~3000	~1200
Dielectric Loss	<0.0076	<0.025	<0.005	<0.0092	<0.06	<0.03
Tunability (%)	68%	70%	51%	3.8%	-	-
$\Delta_{C}/C_{25{}^{\circ}C}$	11.7%	32.6%	37.1%	-	25.6%	6.25%

$\Delta_{C}/C_{25{}^{\circ}C}$: Maximum rate of change in dielectric constant from room temperature.

## Data Availability

The original contributions presented in the study are included in the article, further inquiries can be directed to the corresponding authors.
